# Assessment of the Quality of Saudi Patient Information Leaflets (PILs) Based on the Accuracy of Physical Description and Frequency of Solid Dosage Forms

**DOI:** 10.3390/healthcare10030501

**Published:** 2022-03-09

**Authors:** Turki Al Hagbani, Dareen Alrdaian, Reem Q. Alshammari, Ghaliah Alshammary, Mukhtar Ansari

**Affiliations:** 1 Department of Pharmaceutics, College of Pharmacy, University of Hail, Hail 81442, Saudi Arabia; dareenalrdaian@gmail.com (D.A.); remalzw@gmail.com (R.Q.A.); ghaliahalsh1@gmail.com (G.A.); 2 Department of Clinical Pharmacy, College of Pharmacy, University of Hail, Hail 81442, Saudi Arabia; m.ansari@uoh.edu.sa

**Keywords:** color, community pharmacies, patient information leaflets, physical description, quality

## Abstract

The physical description of dosage forms is one of the most important considerations in avoiding patient confusion and minimizing medication errors. This study aimed to determine the quality and accuracy of the physical descriptions in patient information leaflets (PILs). This cross-sectional study constituted a total of 200 drugs and PILs that were randomly selected, by pharmacy students, from Al-Dawaa community pharmacies in the Hail region of Saudi Arabia, from January 2021 to July 2021. The drugs and PILs were thoroughly evaluated in accordance with the Gulf Cooperation Council (GCC) guidelines for PILs. The accuracy screening of the total solid dosage forms (capsules + tablets) revealed that 45% showed complete accuracy and compliance, 1.5% showed poor accuracy and compliance, and 53% showed no accuracy and no compliance with the GCC guidelines and the Saudi Food and Drug Authority (SFDA) guidelines. In the frequency test, color, shape, and imprints accounted for 95.6%, 79.1%, and 73.6%, respectively. However, the size of the dosage form was the least (2.2%) used physical description feature. These findings recommend pharmaceutical companies to pay more attention to the written physical description in their PILs. Additionally, the process of PIL approval should be optimized, evaluated, and updated systematically to ensure that they contain the essential information.

## 1. Introduction

Medications are part of daily life, and it is always better to have pertinent information about them to minimize medication errors and avoid medicine-related adverse drug reactions. Delivering the correct information to the correct person will offer significant benefits. On the other hand, misinformation can lead to disasters, especially in the case of medications, which are a matter of life and death [1]. To disseminate critical information about drugs and medication, pharmaceutical companies provide patient information leaflets (PILs). PILs are still the most effective and reliable primary source of drug information that patients accept as counselling aids. The most common information provided by PILs includes directions, drug information, safety, and advisories [2,3,4]. According to Nathan et al., 49.2% of patients always read PILs, whereas 21.2% often read PILs. Furthermore, their study indicated that 65% of the patients found PILs to be extremely informative and easy to read and understand, and that patients always or often read the PILs for new drugs [5]. This study highlights that PILs are an effective and reliable method of communication in terms of the proper use of medication.

Medical regulations play a critical role in determining the information presented in PILs. These regulations differ from country to country. In Europe, pharmaceutical manufacturers are required to prepare PILs for newly registered drugs and provide information about the medicine, including a physical description of the medicinal product, such as color, shape, and size [6]. In the USA, PILs are considered the Saudi Food and Drug Authority (FDA)-approved patient labeling, which is required for specific drugs, such as oral contraceptives and estrogen-containing products; however, for other types of medicines, the provision and distribution of PILs are voluntary [7]. In Saudi Arabia, regulations are similar to the European guidelines. Pharmaceutical companies must submit two documents to the Saudi Food and Drug Authority (SFDA). One is a summary of product characteristics (SPC), which provides healthcare providers with information on how to utilize a pharmaceutical product in a safe and effective way. The other is a PIL, which should be developed in accordance with the SPC [8]. Any medication (prescribed or non-prescribed) obtained from pharmacies must be dispensed in its original packaging with a PIL, similar to an SPC [9]. To prepare PILs for the Saudi Arabian market, pharmaceutical manufacturers are required to follow the Gulf Cooperation Council (GCC) guidance for presenting the labeling information, SPC, and PIL, which serves as a template for preparing PILs in Saudi Arabia [8]. The GCC guidelines state that a physical description of the dosage forms is required to be presented in PILs, which is similar to the information required by European guidelines [8]. 

The best way to minimize medication errors and patients being misled is to provide an accurate physical description of medications, which can help patients understand their medications better and avoid medication errors. Such critical information helps patients to identify and distinguish between counterfeit drugs and generic drugs [10]. The UK National Patient Safety Agency and Institute for Safe Medication Practices endorse utilizing physical description items such as color discrimination to help patients differentiate between drugs [11,12].

PILs have long been available on the market and in practice in Saudi Arabia. However, there are several issues, and the way they are presented still has to be improved. A study from Saudi Arabia showed that 17.3% of patients found their PILs had long sentences with a tiny font size that were very difficult to understand [13]. Therefore, a user testing method before the distribution of PILs should be used to assess acceptability, readability, ease of utilization, and understanding of information [2]. To our knowledge, no studies have been conducted in Saudi Arabia to determine the quality and accuracy of the physical descriptions in PILs. This study was designed to fill this gap by evaluating the quality of Saudi PILs and their accuracy regarding physical descriptions, such as color, shape, size, and imprint. In addition, we aimed to identify the most frequent physical description items written in Saudi Arabian PILs.

## 2. Methods

### 2.1. Study Design

This was a community pharmacy-based cross-sectional study.

### 2.2. Study Setting

The study was conducted in Al-Dawaa community pharmacies in the Hail region of Saudi Arabia.

### 2.3. Sample Size, Sampling, and Study Criteria

A total of 200 solid dosage forms were randomly selected from a population of 500 drugs that met the study’s criteria (i.e., availability, solid dosage forms, and easy access with non-destructive method of packing material).

### 2.4. Data Collection and Analysis

The solid dosage form drugs meeting the study criteria were sampled by the pharmacy (PharmD) students from Al-Dawaa community pharmacies in the Hail region of Saudi Arabia. 

The drugs were chosen regardless of brand, manufacturer, or category. Among the solid dosage form drugs, such as tablets and capsules, tablets performed better than capsules in terms of meeting the selection criteria. This cross-sectional study took place from January 2021 to June 2021. The selected drugs were in solid dosage forms manufactured by different pharmaceutical companies, both local and international, and are registered in the SFDA and classified as over-the-counter (OTC) and prescribed medications. Two hundred hard copies of PILs provided by manufacturers were evaluated using accuracy and frequency tests to ensure that the selected drugs and provided PILs covered physical description items. Five important physical description items (color, shape, imprint, size, and score line) were selected and evaluated based on the GCC guidance and new templates from the SFDA (version 2.1 on 1 March 2021) for preparing PILs in Saudi Arabia [14].

In the accuracy test, the evaluation process included matching between the written information in PILs and the accuracy of the physical description presented against the physical state of the drug, as seen by the naked eye. Based on the matching process, data were categorized into three groups. The first group included PILs that showed complete compliance (C.C) with the GCC guidelines, in which three or more physical description items were presented that showed a completely accurate physical appearance matched physically with the relevant drug, as seen by the naked eye. The second group included PILs that showed partial compliance (P.C) with the GCC guidelines, in which one or up to three physical description items were presented and showed poor matching (accuracy) with the physical appearance of the relevant drug. The third group included PILs that showed no compliance (N.C) with the GCC guidelines and no accurate matching or no matching with the physical description of the available drug.

In the frequency test, the evaluation process included the analysis of the most frequent physical description items used, such as color, shape, size, score line, and imprint, both written in PILs and presented in the solid dosage form, with complete adherence for each solid dosage form selected.

## 3. Results

**Figure 1:**
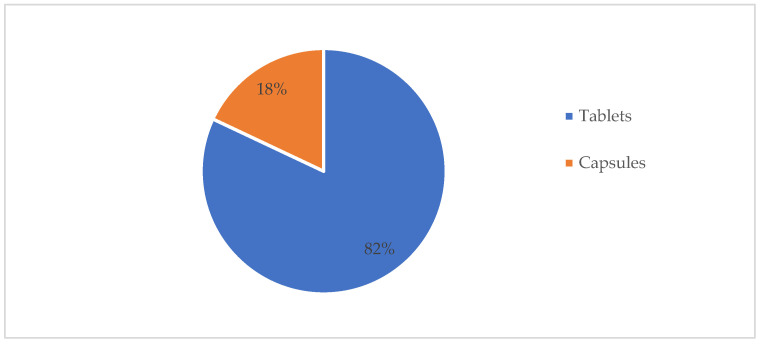
Distribution of the total selected drugs based on the solid dosage forms.

A total of 200 drugs and PILs meeting the study’s criteria were randomly selected and evaluated to assess the quality of Saudi PILs based on their accuracy regarding the physical description of the solid dosage forms and identifying the most frequent physical description items used and written in Saudi PILs. The dosage form distribution for the selected drugs was 82% and 18% for tablets and capsules, respectively (Figure 1). 

Based on the presence or absence of the score line, the tablet dosage form was divided into two groups: scored line tablets, with 39 drugs, and non-scored line tablets, with 126 drugs (Table 1). According to the matching and evaluation results, tablets showed more complete adherence (CA) and non-adherence (NA) than capsules, although partial adherence (PA) was significantly more widespread in capsules than tablets (Table 1). The accuracy of the presence or absence of the score line on tablet dosage forms was also determined. Scored line tablets were observed to show a higher degree of adherence to the GCC PIL requirements for presenting the score line and writing the physical description in PILs compared to tablets that were not scored (Table 1). 

**Figure 2:**
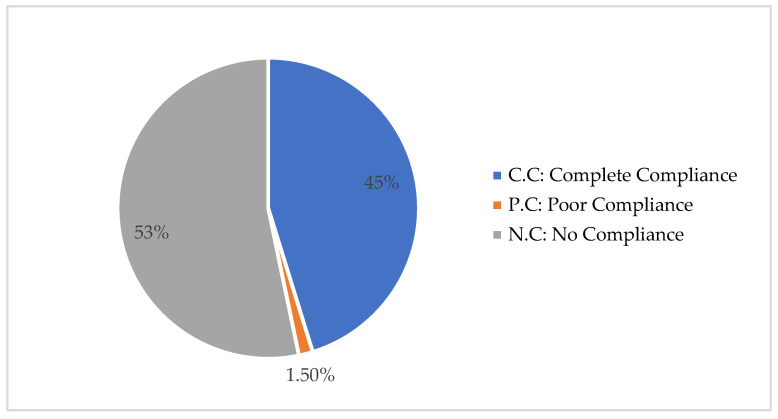
Distribution of the total selected drugs based on the GCC guidance and new templates from the SFDA (version 2.1 on 1 March 2021) for preparing PILs in Saudi Arabia.

The initial accuracy screening of the total solid dosage forms (tablets and capsules) found that 45% belonged to the first group, which showed complete accuracy and compliance with the GCC guidelines, 1.5% belonged to the second group, which showed poor accuracy and compliance with the GCC guidelines, and 53% belonged to the third group, which showed no accuracy and no compliance with the GCC guidelines (Figure 2). 

Only the first group (complete adherence—CA) of all solid dose forms (*n* = 91) was evaluated in a frequency test. Among them, tablets (*n* = 80, 87.9%) and capsules (*n* = 11, 12.1%) only passed the requirements for total adherence; thus, the quantities for capsules are tiny. According to frequency test findings, color, shape, and imprint were the most commonly utilized physical attributes to describe adherent solid dosage forms (tablets and capsules). Size, on the other hand, was the physical description attribute that was used the least, accounting for only 2.2% of the total PILs (Table 2). 

Table 3 depicts the characteristics of pharmaceutical products with adequately written physical descriptions and matching. This comprises three different drugs: pantoprazole, diclofenac sodium, and cetirizine dihydrochloride, all of which had detailed physical descriptions and illustrations in their PILs.

The features of drugs with inadequately worded physical descriptions and matching are shown in Table 4. This covers two separate tablets, captopril and amlodipne, both of which have poorly worded physical explanations that contradict the images. 

The features of pharmaceutical items that failed to fulfill the GCC requirements and matching are shown in Table 5. Three drugs from different categories, namely, mefenamic acid, rosuvastatin, and bisoprolol hemifumarate, are depicted in the table, and their PILs had no physical descriptions. 

Table 6 shows a practical example of a medicine (diclofenac) produced by three separate companies. Two of them explicitly provided physical details in their PILs, whereas the third did not state anything at all.

## 4. Discussion

The main objectives of this study were to assess the accuracy of physical description items and identify the most frequent physical description items written in PILs used in Saudi Arabia. In this study, almost one half (54%) of the evaluated PILs showed a low accuracy of written physical description items. This finding is supported by Sukkari et al., who found that the celecoxib leaflet included only 30% of the required information, whereas the paroxetine and lamotrigine leaflets included 24% and 20%, respectively [15]. In another study, Bawazir et al. discovered that PILs for marketed Saudi drugs contained limited and incomplete information, with certain sections missing [13]. 

In the frequency test, the evaluation was only performed for the first group (complete adherence—CA) of the total solid dosage forms. Evaluation of the PILs found that 95.6% mentioned and focused on the color property for the total solid dosage forms, which might be attributed to the coloration of tablets and capsules that is widely utilized as a tool for identification and enhancing patient adherence. There are certain reasons behind the abundant use of color, as it is the most frequently described feature. Studies have demonstrated the utility of color in the marketing and identification of products. Initially, color is the most critical factor affecting people’s perception of something, so the selection of color can improve or reduce the productivity of any product [16]. For example, one study that involved customers for vitamins packaged in a black container with white lettering showed that they mistook the vitamins for poison because black color is often related to poison in Western culture [17]. Other researchers studied patients’ perception of color in pharmaceutical products and found that patients who took drugs daily preferred brightly colored tablets, and the right selection of color can make an emotional appeal to the patients, which eventually reduces medication errors and increases patients’ compliance with and adherence to their prescription medication plan [18]. Furthermore, color can aid pharmaceutical companies in identifying their products among counterfeit drugs by quantitative measurement of the color on the surface of tablets with a colorimetric technique [10]. Colors also play a vital role in treatment, as ancient Egyptians considered color as a form of therapy [19]. Therefore, colors have several uses and meanings in marketing based on culture, social habits, and geographical background [16].

The second most common physical feature used to describe the solid dosage forms was shape, at 79.1%. Having a unique tablet shape can aid patients in recognizing their drugs and facilitate the swallowing process. For instance, a drug used for controlling hypertension, Concor^®^ (5 mg) by Merck KGaA, Darmstadt, Germany, uses a heart-shaped tablet (Table 5). Generally, the shape also aids patients in the swallowing process because most tablets are circular or have rounded corners [20]. 

The imprint feature was estimated to be the third most common physical description, at 73.6%. Certain pharmaceutical companies manufacture their tablets and capsules with some words, numbers, or even a logo to ease their identification by patients and make medication products more complicated to counterfeit. Therefore, a unique property, such as an imprint with a good and readable quality, also helps patients to differentiate between counterfeit and generic drugs [21] and prevents confusion among other solid dosage forms [18]. 

Scored line tablets showed a higher degree of adherence to the GCC PIL requirements by 60%. This proportion is nevertheless risky because the presence or absence of the score line informs patients indirectly to break, chew, or crush the tablets, despite the fact that the GCC guidelines require all pharmaceutical manufacturers to mention in their PILs whether the tablet is scored or not [8]. Certain tablets are designed as modified release tablets and enteric-coated tablets. As a result, when patients break down these tablets, the release profile mechanism is destroyed. 

Finally, size was used as a descriptive property in the PILs for 2.2% of pills. Size seems to be the least mentioned property for oral tablet or capsule dosage forms because the size description is still challenging; other parameters such as tablet number, peak size, and strength can provide indirect information on the size or volume [22].

To provide a practical example, a generic drug, diclofenac, from three different pharmaceutical companies (local and international) was assessed and evaluated to validate the different levels of quality of Saudi PILs and their accuracy regarding the physical description (Table 6). In this example, we found that two out of three mentioned the color of the tablets and had a good physical description, and one of them mentioned the imprint and score line. For the third product, no physical description items were mentioned in its PIL. Visual characteristics such as color, imprint, shape, and score line are particularly important to include for each pharmaceutical product, as patients typically shift from one generic product to another. Therefore, more efforts from international and local regulatory agents are required to emphasize the physical description of OTC or generic drugs.

The availability and feasibility of the solid dosage forms of products were the limiting factors in this study. Most pharmaceutical products in community pharmacies have opaque blisters, and the physical descriptions are written in their PILs but could not be evaluated and matched with the physical state of the drugs, as seen by the naked eye, with a non-destructive method. A multicenter study comprising more dosage forms, such as semi-solid or liquid forms, would have helped this study to generate more comprehensive data and conclusions. 

## 5. Conclusions

Marketed pharmaceutical solid dosage form drugs exhibited variances in their adherence to the GCC requirements for PILs. Failing the accuracy test was prominent in tablets, particularly non-scored tablets. The most important physical description items used in PILs were color, shape, and imprint, whereas the least used item in physical descriptions of the solid dosage forms was size. A practical example showed the paradox of this issue, even with the same generic drug, diclofenac, from three different pharmaceutical companies (local and international). Based on these findings, we suggest that pharmaceutical companies should focus on using the color, shape, and imprints in their PILs, as this could help patients to identify their drugs, reduce the risk of medication errors, and enhance patients’ awareness to differentiate between generic drugs and counterfeit medications. In addition, the process of approving PILs should be optimized, evaluated, and updated systematically to ensure that they contain the essential information to help patients in the safe use of drugs.

## Figures and Tables

**Table 1:** Summary of the accuracy test results.

Grouping Based on the Solid Dosage Forms Available	Tablets (*n* = 165)	Capsules (*n* = 35)
Group 1 (Complete Adherence—CA)	48.5%	31.4%
Group 2 (Partial Adherence—PA)	1.2%	65.8%
Group 3 (Non-Adherence—NA)	50.3%	2.8%
**Grouping Based on Accuracy Test**	**Scored Tablet (*n* = 39)**	**Non-Scored Tablet (*n* = 126)**
Group 1 (Complete Adherence—CA)	59%	45.3%
Group 2 (Partial Adherence—PA)	5%	0%
Group 3 (Non-Adherence—NA)	35%	54.7%

**Table 2:** Summary of the frequency test results based on the solid dosage forms available.

Variables	Tablets (*n* = 80)	Capsules (*n* = 11)	Total (Tablets + Capsules) (*n* = 91)
Color	95%	100%	95.6%
Shape	86.25%	27.3%	79.1%
Imprint	75%	63.6%	73.6%
Score line	-	-	-
Size	-	18.2%	2.2%

**Table 3:** Characteristics of pharmaceutical products with good written physical descriptions and matching.

No	Product Name (API, Generic Name, Strength)	Written Physical Description in PIL	Image
1	API: Pantoprazole Trade name: Pantomax^®^ Strength: 20 mg Dosage form: Tablet	Oblong, yellow, enteric-coated tablets.	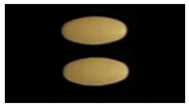
2	API: Diclofenac sodium Trade name: Voltic^®^ Strength: 50 mg Dosage form: Tablet	Light brown-colored, round, bioconvex, enteric-coated tablets engraved with (JP 22) on one side and plain on the other.	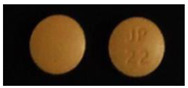
3	API: Cetirizine dihydrochloride Trade name: Zyrtec^®^ Strength: 10 mg Dosage form: Tablet	White, oblong, film-coated tablets with break line and Y-Y logo.	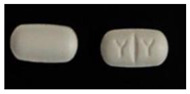

**Table 4:** Characteristics of pharmaceutical products with poorly written physical descriptions and matching.

No	Product Name (API, Generic Name, Strength)	Written Physical Description in PIL	Image
1	API: Captopril Trade name: Acetab^®^ Strength: 50 mg Dosage form: Tablet	White, round tablets. Score line not mentioned.	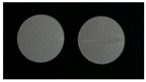
2	API: Amlodipine Trade name: Lodipam^®^ Strength: 5 mg Dosage form: Tablet	Almost-white, octagonal-shaped, film-coated tablets. Score line and tablet imprint not mentioned.	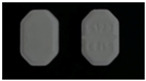

**Table 5:** Characteristics of pharmaceutical products that did not fulfill the GCC guidelines and matching.

No	Product Name (API, Generic Name, Strength)	Written Physical Description in PIL	Image
1	API: Rosuvastatin Trade name: Ivarin^®^ Strength: 10 mg Dosage form: Tablet	No physical description in PIL.	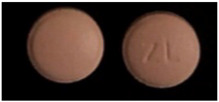
2	API: Bisoprolol hemifumarate Trade name: Concor^®^ Strength: 5 mg Dosage form: Tablet	No physical description in PIL.	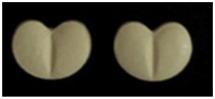
3	API: Mefenamic acid Trade name: Ponstan^®^ Strength: 500 mg Dosage form: Tablet	No physical description in PIL.	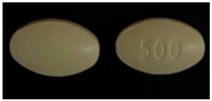

**Table 6:** Practical examples.

No	Product Name (API, Generic Name, Strength)	Written Physical Description in PIL	Image
1	API: Diclofenac Trade name: Emifenac^®^ Strength: 50 mg Dosage form: Tablet	White to off-white, circular tablets with GP11 logo on one side and break line on the other side.	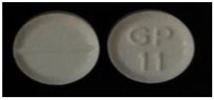
2	API: Diclofenac Trade name: Diclac^®^ Strength: 75 mg Dosage form: Tablet	Round bilayer tablet (white, pink) with plain surface.	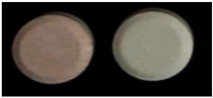
3	API: Diclofenac potassium Trade name: Rapidus^®^ Strength: 25 mg Dosage form: Tablet	No physical description in PIL.	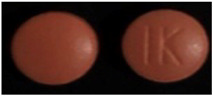

## Data Availability

Not applicable.
